# The Nucleoid Occlusion SlmA Protein Accelerates the Disassembly of the FtsZ Protein Polymers without Affecting Their GTPase Activity

**DOI:** 10.1371/journal.pone.0126434

**Published:** 2015-05-07

**Authors:** Elisa J. Cabré, Begoña Monterroso, Carlos Alfonso, Alicia Sánchez-Gorostiaga, Belén Reija, Mercedes Jiménez, Miguel Vicente, Silvia Zorrilla, Germán Rivas

**Affiliations:** 1 Centro de Investigaciones Biológicas, Consejo Superior de Investigaciones Científicas (CSIC), Madrid, Spain; 2 Centro Nacional de Biotecnología, Consejo Superior de Investigaciones Científicas (CSIC), Madrid, Spain; 3 Instituto de Química-Física Rocasolano, Consejo Superior de Investigaciones Científicas (CSIC), Madrid, Spain; University of Groningen, Groningen Institute for Biomolecular Sciences and Biotechnology, NETHERLANDS

## Abstract

Division site selection is achieved in bacteria by different mechanisms, one of them being nucleoid occlusion, which prevents Z-ring assembly nearby the chromosome. Nucleoid occlusion in *E*. *coli* is mediated by SlmA, a sequence specific DNA binding protein that antagonizes FtsZ assembly. Here we show that, when bound to its specific target DNA sequences (SBS), SlmA reduces the lifetime of the FtsZ protofilaments in solution and of the FtsZ bundles when located inside permeable giant vesicles. This effect appears to be essentially uncoupled from the GTPase activity of the FtsZ protofilaments, which is insensitive to the presence of SlmA·SBS. The interaction of SlmA·SBS with either FtsZ protofilaments containing GTP or FtsZ oligomers containing GDP results in the disassembly of FtsZ polymers. We propose that SlmA·SBS complexes control the polymerization state of FtsZ by accelerating the disassembly of the FtsZ polymers leading to their fragmentation into shorter species that are still able to hydrolyze GTP at the same rate. SlmA defines therefore a new class of inhibitors of the FtsZ ring different from the SOS response regulator SulA and from the moonlighting enzyme OpgH, inhibitors of the GTPase activity. SlmA also shows differences compared with MinC, the inhibitor of the division site selection Min system, which shortens FtsZ protofilaments by interacting with the GDP form of FtsZ.

## Introduction

Bacterial division in *E*. *coli* is achieved by the formation of the divisome, a multiprotein complex comprising over 10 essential proteins [1,2]. A central component of this system is FtsZ, a widely conserved GTPase that assembles into a ring-like structure anchored to the bacterial membrane by interaction with ZipA and FtsA, the other two proto-ring proteins (reviewed in [3,4]). The proto-ring serves as a scaffold for the recruitment of the remaining downstream division proteins into the division ring, located at midcell by the action of two negative regulators of FtsZ assembly: the Min system and the nucleoid occlusion (reviewed in [3,4]).

The GTP induced FtsZ polymerization and its regulation have deserved particular attention in the field of bacterial division, given their relevance for the whole process and their high level of complexity [5,6]. FtsZ polymers are arranged into a number of structures of different complexity depending on the polymerization conditions [3,5,7–10]. At neutral pH, moderate concentration of KCl, and Mg^2+^ concentrations between 0.3 and 5 mM, FtsZ polymerizes into single stranded protofilaments narrowly distributed in size following a cooperative mechanism [10–12]. These polymers present a fast protein turnover rate and disassemble when the GDP/GTP ratio in the solution or in the cells increases upon GTP hydrolysis [13]. The polymers can be stabilized at steady state while preserving their dynamic properties with a GTP regeneration system [14]. Polymerization of FtsZ for longer times has been also achieved in the presence of Guanosine-5'-[(α,β)-methyleno]triphosphate (GMPCPP), a slowly hydrolysable analog of GTP [5]. The size of the GTP induced FtsZ protofilaments, ranging from 30 to 100 FtsZ units depending on the precise buffer conditions [11,13,15–19], is too small to encompass the whole perimeter of *E*. *coli* midcell. Two different models were proposed to describe how these protofilaments might assemble to form the Z-ring. Cryo-EM tomography images of *Caulobacter* pointed to a staggered overlapping where the subunits are scattered around the circumference of the cell [20], while AFM suggested annealing into one or a discrete number of longer protofilaments [21]. Recent high-resolution microscopy studies suggest that the Z-ring is actually composed of short FtsZ filaments [22]. Although their precise arrangement to produce the functional Z-ring structure remains to be determined, it seems clear that the crowded nature of the bacterial cytoplasm influences their organization, as demonstrated by the presence of substantially larger structures arising from protofilament bundling in solutions containing macromolecular crowding agents such as Ficoll 70 [8].

Together with the Min system, nucleoid occlusion is a negative regulatory mechanism that controls Z-ring assembly in *E*. *coli* preventing divisions at non-central regions of the bacteria or over the nucleoid. The Min system consists of a complex of three proteins (MinC, D and E) [4,23]. MinC is the protein of this system that directly interacts with FtsZ interfering with its assembly [24]. This protein is anchored to the membrane through MinD, which further activates its inhibitory function [25]. MinE directs a pole-to-pole oscillatory behavior of the interacting MinCD creating a gradient such that the local concentration of inhibitor is lowest at midcell, favoring FtsZ assembly in this region [4,23,26]. While the role of the Min system in division has been extensively studied in solution, under cell-like conditions *in vitro* and in live bacteria (see, for instance, [24,27–30]), the number of reports on the mechanism by which nucleoid occlusion modulates Z-ring assembly is comparatively lower [31–35]. Indeed the nucleoid occlusion factors, SlmA in *E*. *coli* and Noc in *B*. *subtilis*, were identified less than 10 years ago [36–38].

It has been recently shown that SlmA is a sequence-specific DNA binding protein recognizing a number of natural sequences called SlmA-binding sequences (SBSs) [31,32]. Analysis of the effect of SlmA and of its specific nucleoprotein complexes on FtsZ assembly indicates that SlmA (as a dimer or a higher order oligomer) blocks FtsZ polymerization [31]. Early studies alternatively proposed that SlmA SBS complexes do not disrupt FtsZ protofilaments but rather induce the formation of structures consistent with an antiparallel orientation of the FtsZ protofilaments, which would interfere with Z-ring formation [32,39]. After the recent demonstration of the inhibition of FtsZ assembly by SlmA under a variety of buffer conditions [35] this other mechanism seems unlikely. In this study, Du and Lutkenhaus found that SlmA interacts with FtsZ through its conserved C-terminal tail, a central hub targeted by other division proteins like MinC and the proto-ring proteins FtsA and ZipA [35]. The effect of the SlmA SBS complexes on the GTP hydrolysis rate of FtsZ remains unclear, as independent reports have shown either a significant enhancement [31] or an insensitivity [33] of the GTPase activity of FtsZ upon interaction with SlmA SBS complexes. The antagonistic activity of SlmA has been reported to be increased by the interaction with specific SBS sequences [31], probably due to a conformational change in the protein that leads to the full exposure of its FtsZ binding sites [34]. According to the crystal structure of the SlmA DNA complex, the protein binds to the DNA as a dimer of dimers and this interaction locks the flexible Helix-Turn-Helix domain of SlmA into a single conformation [33]. Moreover, binding of SlmA induces deformations at each side of the SBS, which probably allows the recruitment of additional SlmA molecules [33].

Here we have quantitatively analyzed the effect of SlmA on FtsZ assembly into protofilaments both in the presence and absence of its specific SBS sequences by using biophysical and biochemical approaches. The complexes of SlmA with three different SBS sequences were analyzed and the interaction of the SlmA SBS complexes with FtsZ-GTP protofilaments and FtsZ-GDP oligomers assessed. We have also studied the effect of SlmA, free or in complex with the DNA, on FtsZ bundles reconstructed in cell-like permeable giant unilamellar vesicles (GUVs). This work provides new data on the regulation of FtsZ assembly by the active nucleoprotein complex of SlmA through a mechanism different from those proposed for other antagonists, namely SulA and OpgH. Some differences and similarities with respect to the inhibition by MinC are also described.

## Materials and Methods

### Protein expression, purification and labeling

*E*. *coli* FtsZ was isolated as described elsewhere [40]. SlmA was purified and cleaved from SUMO protein essentially as described in [31]. The plasmid encoding SlmA tagged with SUMO (pTB147 [31]) was a kind gift of Thomas Bernhardt (Harvard Medical School, Boston, MA).

FtsZ was labeled in the amine groups with Alexa Fluor 488 succinimidyl ester dye (Molecular probes, Invitrogen) in its assembled form as previously described [8,41]. SlmA was labeled with Alexa Fluor 488 or Alexa Fluor 647 succinimidyl ester dyes (SlmA-Alexa 488 and SlmA-Alexa 647). The reaction was allowed to proceed for 1 h. at RT using a SlmA:dye molar ratio of 1:8 and the free dye was subsequently separated from the labeled protein by size exclusion chromatography. The labeling ratio of the two proteins, calculated from the molar absorption coefficients of the proteins and the dyes, ranged between 0.2–0.9 moles of dye per mole of protein.

### DNA hybridization

Single stranded oligonucleotides (IBA GmbH) were purchased either unlabeled or labeled with fluorescein using the phosphoramidite chemistry. Hybridization of complementary strands was conducted on a thermocycler by heating the mixture to 85–95°C and slowly cooling down to room temperature. Hybridizing reactions with a labeled oligonucleotide contained a 10% excess of the unlabeled oligonucleotide in the mixture. The SBS sequences used in this work were SBS17 (5’-TCAAC**GTTAGTGACCATTTACTTAC**TTTTG-3’), SBS consensus sequence (SBScons, 5’-AA**GTAAGTGAGCGCTCACTTAC**GT-3’) and SBS12 (5’-CG**CGAAGTGAACGCTAACTCAC**TG -3’) [31]. The bases recognized by SlmA are marked in bold. For SBS12 and SBScons the fluorescein dye, when present, was bound to the 5’ end of the sense strand whereas for the SBS17 the dye was attached to the T base at position 27 from the 5’ end of the antisense oligonucleotide (underlined above). Proper hybridization was checked by polyacrylamide gel electrophoresis and ethidium bromide staining.

### Production of giant unilamellar vesicles

SlmA, SBScons and FtsZ containing GUVs made of egg-yolk L-α-phosphatidylcholine (EPC, Avanti Polar Lipids, Alabaster, AL) were prepared according to a water-in-oil double emulsion method recently optimized [42]. Vesicles were made permeable by mixing, prior to lipid film formation, EPC with 10 mol% of 1,2-dimyristoyl-sn-glycero-3-phosphocholine (DMPC, Avanti Polar Lipids, Alabaster, AL) that induces heterogeneities in the vesicle membrane at temperatures above its Tm (23°C [43]) through which small ligands can diffuse. External addition of MgCl_2_ (3–4 mM) and GTP (2 mM) triggered the polymerization of FtsZ (12 μM) into bundles inside the GUVs. The protein was equilibrated in 50 mM Tris-HCl pH 7.5 containing 300 mM KCl, 100 mM sucrose and 50 g/L Ficoll 70. When present, 1–50 μM SlmA, 0.2–2 μM SBScons and 1.2 μM FtsZ-Alexa 488 were added to this solution. Visualization of precipitated GUVs was conducted on a Leica TCS SP5 AOBS inverted confocal microscope (Leica, Mannheim, Germany) as previously described [42].

### Fluorescence anisotropy binding titrations

Anisotropy experiments were performed on a PC1 photon counting spectrofluorometer (ISS) using 3x3 mm quartz cuvettes (Hellma Hispania). The anisotropy of the samples was repeatedly measured and the average of 10 measurements obtained after reaching equilibration was calculated. Reported anisotropy values correspond to the average of, at least, 3 independent experiments. The temperature was regulated at 20°C and the buffer was 50 mM Tris-HCl, 300 mM KCl, 2 mM MgCl_2_, pH 7.5 (working buffer), unless otherwise stated. The concentration of labeled SBS in the anisotropy titrations with SlmA was 10 nM. The concentrations of SlmA and of fluorescein labelled SBScons in the titrations with FtsZ were 2 and 0.2 μM or 1 μM and 10 nM, respectively. In these experiments, polymerization was elicited by 1 mM GTP and the protofilaments were stabilized by an enzymatic GTP-regeneration system (RS, 2 units/mL acetate kinase and 15 mM acetyl phosphate) [8].

The anisotropy binding isotherms were analyzed with BIOEQS software to retrieve the free energy of formation of the complexes from their individual components using a Marquardt-Levenberg algorithm [44]. The same software was used to assess the errors associated to the free energy values by rigorous confidence limit testing at the 67% and to calculate the amount of SlmA SBS complex from the initial concentrations of SlmA and SBS in the solutions. The binding isotherms of the interaction between SlmA SBS complexes and FtsZ-GTP were analyzed using a 1:1 model compatible with the experimental data to retrieve an apparent *K*_*d*_. This apparent *K*_*d*_ may be corrected by the size of the FtsZ protofilaments, following the reasoning described in detail for analysis of the interaction of ZipA inserted in nanodiscs with FtsZ polymers [45], with the assumption that most FtsZ is in polymeric form and only one SlmA SBS was bound to each polymer along the titrations. All *∆G* values indicated correspond to the dissociation reactions.

### Analytical ultracentrifugation

Sedimentation velocity runs were carried out at 48,000 rpm and 20°C in an XL-I analytical ultracentrifuge (Beckman-Coulter Inc.) equipped with UV-VIS and Raleigh interference detection system, using an An50Ti rotor and 12-mm double sector centerpieces. The experiments were performed in working buffer. In the experiments aimed at determining the stoichiometry of the SlmA SBS complexes, the concentration of SBScons or SBS17 was 1 μM and that of SlmA 5–15 μM. The concentrations of SlmA, SBScons and FtsZ used to measure the interaction of SlmA SBS complexes with FtsZ-GDP were 15 μM, 4 μM and 30 μM, respectively. A tracer amount of FtsZ-Alexa 488 (6 μM) was also included. The sedimentation coefficient distributions were calculated by least squares boundary modeling of sedimentation velocity data using the *c*(*s*) method [46] as implemented in SEDFIT. Calculated *s* values were corrected to standard conditions (water, 20°C, and infinite dilution) using SEDNTERP [47].

### Size exclusion chromatography coupled to multi-angle static light scattering

MALLS measurements were carried out in a DAWN-EOS multi-angle light scattering photometer (Wyatt Technology Corp, Santa Barbara) equipped with an Optilab rEX differential refractometer configured to collect data in parallel from the incoming sample stream, eluting from a coupled dextran-agarose column (Superdex 200 10/300 GL, Pharmacia Biotech, flow rate 0.5 mL/min) at 20°C in working buffer. Solutions were freshly prepared and degassed by centrifugation at 60000 × *g*, 30 min and 200 μL were injected. The concentration of SlmA was 10 μM, and that of the SBScons, 2 μM. The acquired raw data consisted of the scattering intensity at fourteen scattering angles and the differential refractive index of the species fractionated in the column. The Rayleigh ratio was determined from the raw scattering intensity and the w/v concentration calculated from the differential refractive index as described in [48,49]. Raw data were acquired using ASTRA (V.4.90, Wyatt Technology) and exported as text files for subsequent processing and modeling using user-written scripts and functions in MATLAB (Ver. 7.3, MathWorks, Natick, MA). The average molecular masses were calculated from the ratio of scattering to concentration at the peak maximum.

### Fluorescence correlation spectroscopy

FCS experiments were conducted on a Microtime 200 instrument (PicoQuant) with two-photon excitation as previously described [41]. In the measurements, 120 nM FtsZ labeled with Alexa 488 (FtsZ-Alexa 488) was included and additional unlabeled FtsZ was added up to 12 μM. Polymerization was triggered with GTP (1–2 mM) and the protofilaments, even when not specifically stated, were stabilized by the enzymatic GTP-regeneration system. When present, the concentrations of SlmA and SBS were 10 μM and 2 μM, respectively. All samples were incubated for at least 20 minutes in working buffer prior to measurements. The autocorrelation profiles were analyzed by fitting the models described in detail elsewhere [10,41]. Briefly, profiles corresponding to unassembled FtsZ were fit using a two-component model including a fast component, assigned to the free dye, whose diffusion time was independently measured and fixed in the analysis. A three component model was fit to the curves obtained for samples containing FtsZ protofilaments, involving the free dye, the unassembled protein and the protofilament. The diffusion times of the unassembled protein and of the free dye, as well as the contribution of the latter, were constrained in the analysis to the values independently measured in samples lacking protofilaments.

### Electron microscopy

Samples containing FtsZ (12 μM) with or without SlmA (10 μM) and SBS (2 μM) were incubated 15 min at RT in working buffer. 5 min after the addition of 1 mM GTP, samples were diluted 10 times, grids floated on top of the sample solutions for 1 min, blotted and stained for 1 min with 2% uranyl acetate. Images were recorded with a TemCam-F416 CMOS camera (TVIPS) coupled to a JEOL-1200 electron microscope operated at 90 kV.

### 90° light scattering

Measurements were performed on a PC1 photon counting steady-state spectrofluorometer (ISS) at 350 nm and 20°C, in working buffer, using 3x3 mm quartz cuvettes (Hellma Hispania). FtsZ concentration was 12 μM. The elapsed time between addition of the last component in the mixture and the beginning of data collection was 45–90 s. The concentration of GTP was 1 mM.

### GTPase activity

GTPase activity was determined in working buffer and in GUVs encapsulation buffer by measuring released inorganic phosphate with the malachite green-molybdate reagent [50,51]. The concentration of FtsZ was 5 μM and at least two independent measurements were performed in the absence and presence of SlmA (10 μM) with or without SBScons (2 μM).

## Results

### Control by ionic strength of SlmA SBS complex formation

**Fig 1:**
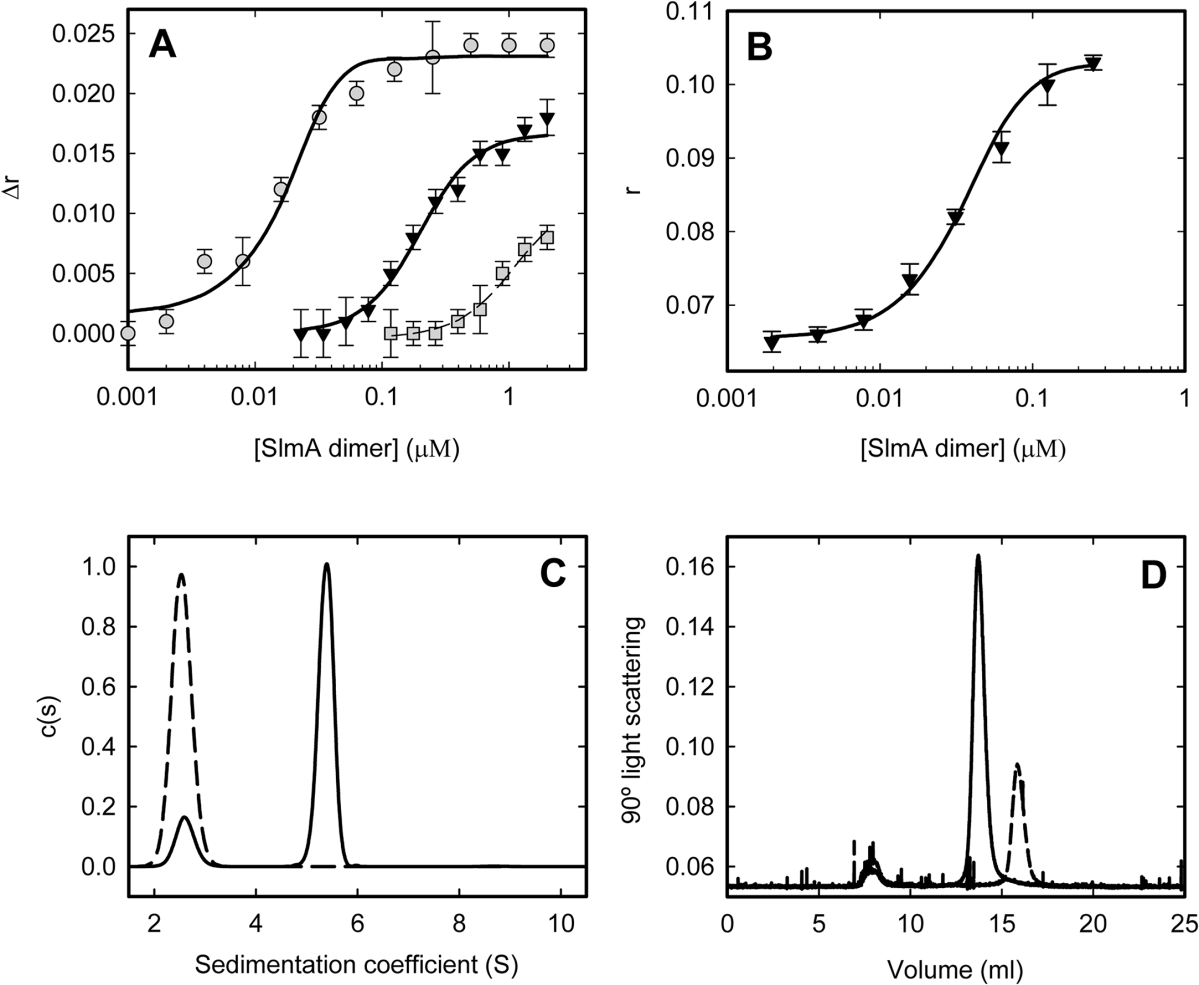
Energetics and stoichiometry of SlmA binding to SBSs. *A*. Dependence of the anisotropy change (*∆r*) of fluorescein labeled SBS17 with SlmA concentration in working buffer (300 mM KCl, triangles) or working buffer but with 100 mM KCl (circles) or 500 mM KCl (squares). *B*. Dependence of the anisotropy (*r*) of fluorescein labeled SBScons with SlmA concentration in working buffer with 300 mM KCl. Isotherms in A and B are the average of three independent experiments ± SD. Solid lines correspond to the fit of the model involving a complex with two SlmA dimers bound to SBS described in the main text, rendering the best fit values in **Table 1**. Dashed line is only aimed to guide the eye. Experiments were conducted at 20°C with 10 nM fluorescein labeled SBS. Excitation and emission wavelengths were 495 nm and 520 nm, respectively. *C*. Sedimentation velocity profiles of 1 μM SBScons in the absence (dashed line) or presence of SlmA (5 μM, solid line). *D*. SEC-MALLS profiles of SlmA (10 μM) in the absence (dashed line) or presence of SBScons (2 μM, solid line). Reactions in *C* and *D* in working buffer (with 300 mM KCl).

In previous studies we have shown that FtsZ polymerizes into single stranded protofilaments, of different size depending on KCl content, in nearly neutral pH buffer with 0.5–5 mM MgCl_2_ and 0.1–0.5 M KCl [10,11]. Since the structure and properties of FtsZ polymers are extremely sensitive to buffer composition [5,6], we decided to use experimental conditions falling within the mentioned ranges to investigate the effect of SlmA and of its nucleoprotein complexes specifically on single stranded protofilaments. Because protein/DNA interactions are generally sensitive to ionic strength we selected the KCl concentration for our study by evaluating the SlmA interaction with a representative 20-bp SBS sequence, SBS17 [31], at different concentrations of this salt. A remarkable effect of ionic strength on the affinity of SlmA for SBS17 was found by anisotropy binding titrations of the labeled oligonucleotide with the protein (**Fig 1A**), indicating a high contribution of electrostatic interactions in the recognition process. At 500 mM KCl the binding did not reach saturation at the highest concentration of SlmA in the assay (4 μM in monomer units, **Fig 1A**). We decided, therefore, to pursue our study at 300 mM KCl, already suggested as a suitable concentration to conduct studies on the interactions of *E*. *coli* division proteins [5].

The stoichiometry of nucleoprotein complexes may vary with buffer conditions and with the particular SBS sequence. Hence, we determined the stoichiometry of the SlmA DNA complexes in our working buffer and with the SBS sequences we selected by analytical ultracentrifugation and SEC-MALLS, in order to quantitatively analyze the anisotropy binding isotherms using suitable stoichiometric models. Our results indicate the formation of a complex composed of four SlmA monomers (or two dimers) per DNA unit (disregarding the SBS) at 300 mM KCl. Sedimentation velocity analysis indicated the formation of a single complex between SBScons and SlmA (**Fig 1C**). Analogous results were obtained for the SBS17 and SBS12 sequences (not shown). The mass of the SlmA SBScons complex determined by SEC-MALLS was (119 ± 5) x 10^3^ (main peak in **Fig 1D**), compatible with a 4:1 complex. This is the same stoichiometry previously determined by crystallography under different conditions using other SBS sequences [33]. In the absence of DNA, SlmA was found to be a dimer according to SEC-MALLS at 10 μM SlmA (mass of (48 ± 2) x 10^3^) and even at 6 μM.

SlmA displays higher affinity for the SBScons sequence than for the other two sequences, being only slightly tighter for SBS17 than for SBS12. The affinity of the interaction of SlmA with SBS was obtained by fitting a model involving a single complex harboring two SlmA dimers per SBS molecule to the anisotropy traces (**Fig 1**and **Table 1**). This is the simplest model compatible with the experimental data. The energetic parameters retrieved from the analysis allowed the calculation of the amount of 4:1 SlmA:SBS complex for different initial concentrations of protein and DNA, under the working conditions of this study.

**Table 1:** Energetic parameters of the interaction of SlmA with SBSs.

DNA	*∆G* ^*a*^ (kcal/mol)	*K*_*d*,*app*_ ^*b*^ (nM)
**SBS17** ^*c*^	18.0 ± 0.2	190 ± 30
**SBScons**	20.3 ± 0.2	25 ± 4
**SBS12**	17.6 ± 0.2	270 ± 50

^*a*^ *∆G* values correspond to the dissociation of the complex with two SlmA dimers bound to the SBS. In 50 mM Tris, 300 mM KCl, 2 mM MgCl_2_, pH 7.5.

^*b*^ *K*_*d*,*app*_, in dimer units, is an apparent *K*_*d*_ calculated from the value of *∆G* divided by 2 and represents the concentration at which 50% of binding occurs.

^*c*^In working buffer with 100 mM KCl, *∆G* for the interaction of SlmA with SBS17 was 21.6 (-0.6,+1.1) kcal/mol, assuming a complex with two dimers.

Uncertainties were calculated by rigorous confidence limit testing at the 67% confidence level, using BIOEQS software.

### SlmA blocks FtsZ-GTP protofilament formation only when bound to SBS

**Fig 2:**
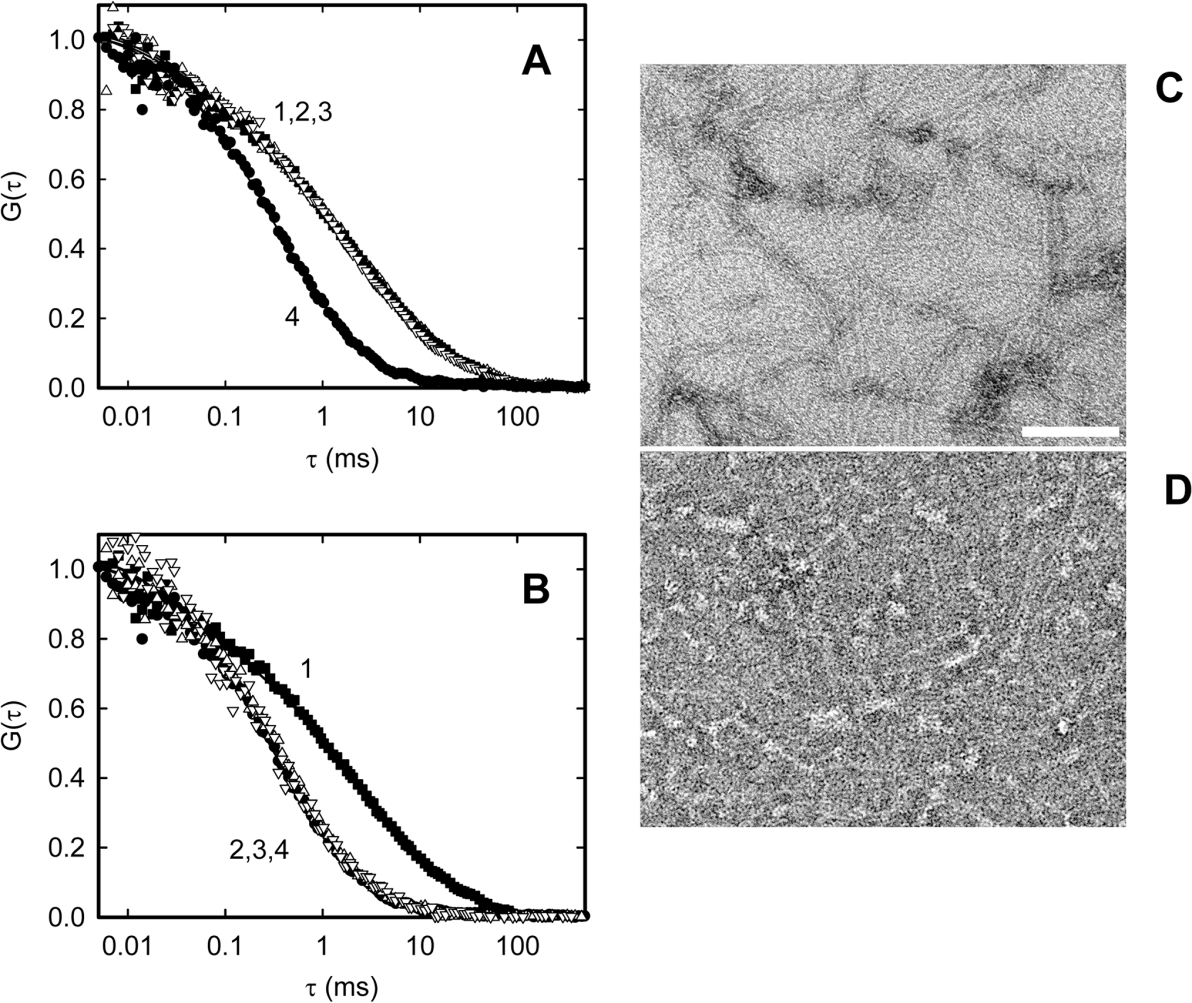
Effect of free or SBS bound SlmA on FtsZ-GTP protofilaments as measured by FCS and EM. *A* and *B*. Normalized FCS autocorrelation profiles of FtsZ-GTP (12 μM) in the presence of free SlmA (10 μM, *A*) and SlmA:SBScons (10 μM:2 μM, *B*). In both panels, curves 2 and 3 indicate SlmA (with or without SBS) added before and after FtsZ polymerization, respectively. Traces corresponding to FtsZ-GTP polymers (curve 1) or FtsZ-GDP (curve 4) are shown for reference. Lines represent best fits of the models indicated in the Materials and Methods section. FtsZ-Alexa 488 (120 nM) was added as a tracer. Measurements were performed in working buffer at 21°C and RS was always added together with GTP. *C* and *D*. Representative micrographs of FtsZ-GTP polymers (12 μM) in the absence (*C*) or presence of the complex formed by SlmA and SBScons (10 and 2 μM, respectively; *D*). Samples were equilibrated in working buffer and polymerization was triggered by the addition of 1 mM GTP. The bar represents 90 nm.

Previous studies have shown that the presence of SlmA results in either disassembly of FtsZ polymers [31,35] or modification of their higher order assembly properties [36] without affecting the ability of the protein to form protofilaments [32,33]. To analyze the effect of free SlmA and SlmA SBS complexes on FtsZ assembly into protofilaments we used FCS. When not in complex with SBS, SlmA had no effect on the polymerization of FtsZ induced by GTP. Protofilaments elicited by addition of GTP in the presence of SlmA showed a diffusion coefficient of around 5 μm^2^s^-1^, in good agreement with that previously determined for FtsZ protofilaments at 300 mM KCl [11], and considerably slower than that of the unassembled protein (*D* ~45 μm^2^s^-1^, **Fig 2A and 2B**). Addition of SlmA to already formed FtsZ protofilaments did not affect their diffusion either, as indicated the superimposition of their autocorrelation profiles with those obtained for the assembled protofilaments in the absence of SlmA (**Fig 2A**). Previous incubation of the samples for 20–60 min did not modify this result.

On the contrary, polymerization of FtsZ with GTP was negatively affected by the SlmA protein when SBS was present. In the presence of SlmA SBS and GTP, no FtsZ protofilaments were detected (**Fig 2B**). Addition of the SlmA SBScons complex, before or after triggering polymerization with GTP, showed that the effect of specific nucleoprotein complexes of SlmA was similar both on the growing FtsZ protofilaments and the already assembled ones (**Fig 2B**). Analogous results were obtained when SBS12 or SBS17 were forming the complex (not shown).

EM analysis of FtsZ polymers in the presence of SlmA SBS also revealed a clear negative influence of the nucleoprotein complex upon FtsZ polymerization into protofilaments, not detected in the absence of SBS even at SlmA concentrations as high as 10 μM. The EM images acquired in the presence of SlmA were similar to those obtained for FtsZ protofilaments in the absence of the protein (not shown). In contrast, when SlmA and SBScons were added to the mixture, the images were essentially devoid of protofilaments (**Fig 2C and 2D**).

### SlmA SBS shortens the lifetime of protofilaments and does not modify the GTPase activity of FtsZ

To better understand the effect of the nucleoid occlusion factor on FtsZ polymerization, the disassembly of FtsZ protofilaments was studied by 90° light scattering in the presence or absence of the SlmA SBS complexes.

**Fig 3:**
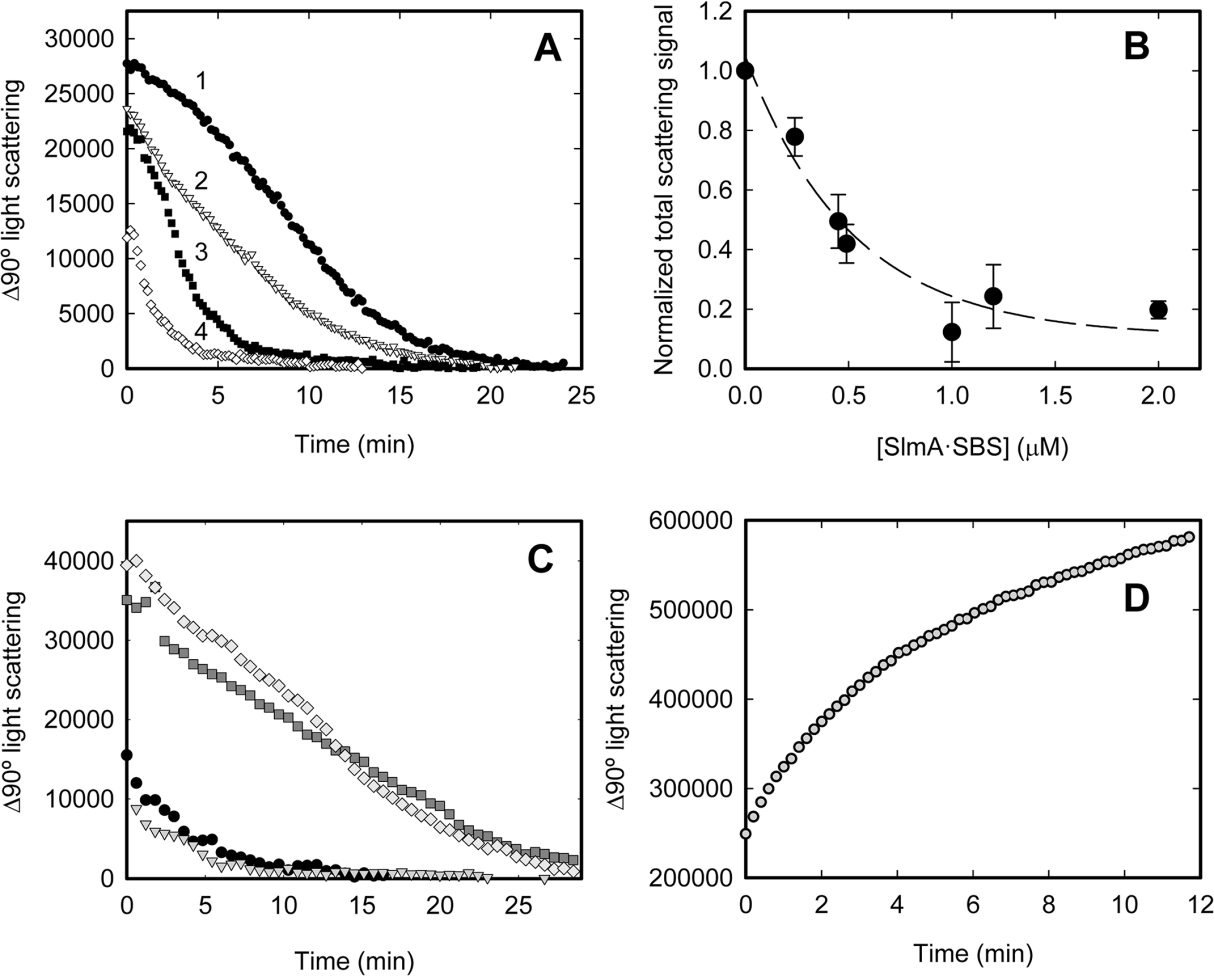
Depolymerization of FtsZ-GTP protofilaments in the absence and presence of SlmA or SlmA SBS and behavior of FtsZ-GMPCPP polymers monitored by static light scattering at 90°. *A*. Representative depolymerization profiles of FtsZ (12 μM) in the absence (trace 1) or presence of SlmA:SBScons 2:0.25 (trace 2), 5:0.5 (trace 3) and 5:1 μM (trace 4). *B*. Dependence of the normalized total scattering signal on the concentration of the SlmA SBS complex, calculated using the binding parameters in **Table 1**and BIOEQS software. The area under the scattering curves shown in *A* was calculated and normalized with respect to that obtained in the absence of SlmA SBS (trace 1). Values shown represent the average of at least 3 independent measurements ± SD and dashed line is only intended to guide the eye. *C*. Depolymerization traces in the absence (grey diamonds) or presence of SlmA (10 μM, dark grey squares), and those obtained upon addition of SlmA:SBScons (10 μM:2 μM) before (solid circles) or after triggering FtsZ polymerization with GTP (grey triangles). *D*. Evolution of the 90° static light scattering signal of FtsZ-GMPCPP polymers (12 μM FtsZ, 0.4 μM GMPCPP) in the presence of SlmA SBScons (10 μM:2 μM). Experiments were conducted in working buffer at 20°C.

Addition of GTP to FtsZ resulted in a boost in the scattering signal because of FtsZ polymerization into protofilaments (**Fig 3A**). In the absence of a system to regenerate GTP, this signal decreased with time due to the disassembly of FtsZ. The FtsZ protofilaments disassembled faster in the presence of SlmA SBScons (**Fig 3A**) and the extent of the acceleration of FtsZ disassembly was found to be dependent on the amount of functional SlmA SBS complex consisting of a dimer of dimers of SlmA bound to SBS. The concentration of this complex was calculated from the initial concentration of its elements, SlmA and SBS, using the energetic parameters experimentally determined from analysis of the interaction (see above and **Table 1**). We observed that different combinations of loading concentrations of SlmA and SBS rendering the same amount of the complex involving four SlmA monomers antagonized FtsZ polymerization at the same level.

The destabilization along time of the protofilaments was detectable at concentrations of the functional nucleoprotein complex as low as 0.25 μM and the global effect was almost complete at 1–2 μM. This last concentration is 5–10 times lower than that of FtsZ (12 μM) (**Fig 3B**). Addition of the nucleoprotein complexes before or after triggering FtsZ polymerization with GTP had the same effect on the lifetime of the protofilaments (**Fig 3C**). As observed in the FCS and EM experiments, no significant changes in the behavior of FtsZ protofilaments were detected in the presence of free SlmA by light scattering (**Fig 3C**).

The reduction of the FtsZ polymers lifetime in the presence of SlmA SBS was observed even in solutions containing the enzymatic GTP-regeneration system. This precluded further characterization to determine the size of the protofilaments severed by SlmA SBS using a combination of biophysical approaches [52], as previously done for MinC disturbed FtsZ polymers [53]. Stabilization of the polymers for their characterization by addition of GMPCPP was not suitable in this case. The presence of SlmA SBS in FtsZ samples containing this nucleotide resulted in a dramatic increase in the 90° light scattering signal (**Fig 3D**), compatible with the previously suggested enhancement of filament bundling under these conditions [35].

**Fig 4:**
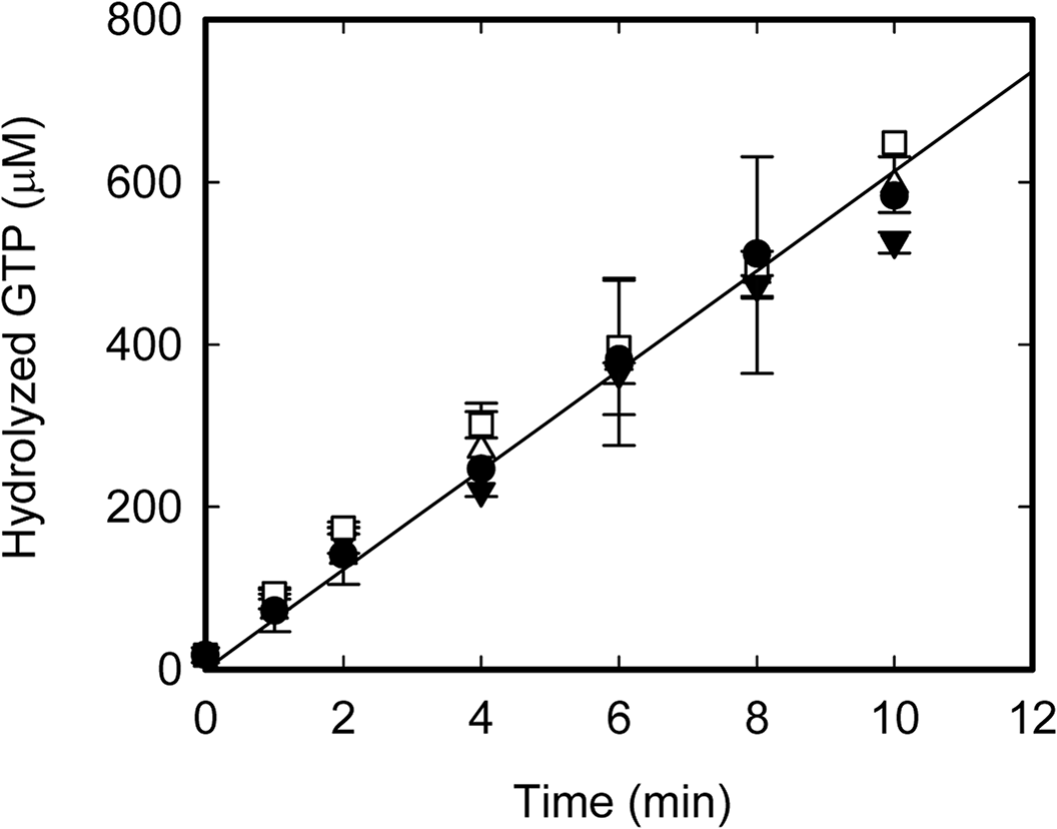
Effect of SlmA and of its nucleoprotein complex on the GTPase activity of FtsZ protofilaments. Triangles up, solid circles, triangles down and squares correspond to FtsZ in the absence and presence of SBScons, SlmA and SlmA SBS, respectively. The GTPase activity was ~12 moles of hydrolyzed GTP/mole of FtsZ/min. The concentration of FtsZ was always 5 μM and, when present, SlmA and SBScons were added at 10 and 2 μM, respectively. Measurements were performed in working buffer.

The faster disassembly of FtsZ protofilaments promoted by the SlmA SBS complexes could be, in principle, a trivial consequence of the initially suggested increase of the GTP hydrolysis rate of FtsZ in the presence of the nucleoprotein complexes [31]. However, our measurements of the GTPase activity of FtsZ showed that it is not significantly changed by the presence of free SlmA or of the specific nucleoprotein complex (**Fig 4**), in good agreement with recent results [33]. These findings contrast with those of Cho *et al*. [31], who found a slight enhancement of the GTPase activity in the presence of SlmA SBS. Notably, we also observed an increase of the GTPase activity under the experimental conditions used by Cho *et al*. (not shown and [31]). The discrepancies might arise from the lower pH and higher Mg^2+^ concentration used in this work that may favor the formation of bundles [50]. The action of SlmA complexes on the bundles would increase the number of loose protofilaments with higher GTP hydrolysis rate compared to the bundles [8,50]. In support of this hypothesis, the other published measurements in which no significant change of the GTPase activity was obtained were performed, as our work, under conditions favoring the formation of single stranded FtsZ protofilaments [33].

### The SlmA SBS complexes interact with both FtsZ-GTP protofilaments and FtsZ-GDP oligomers

**Fig 5:**
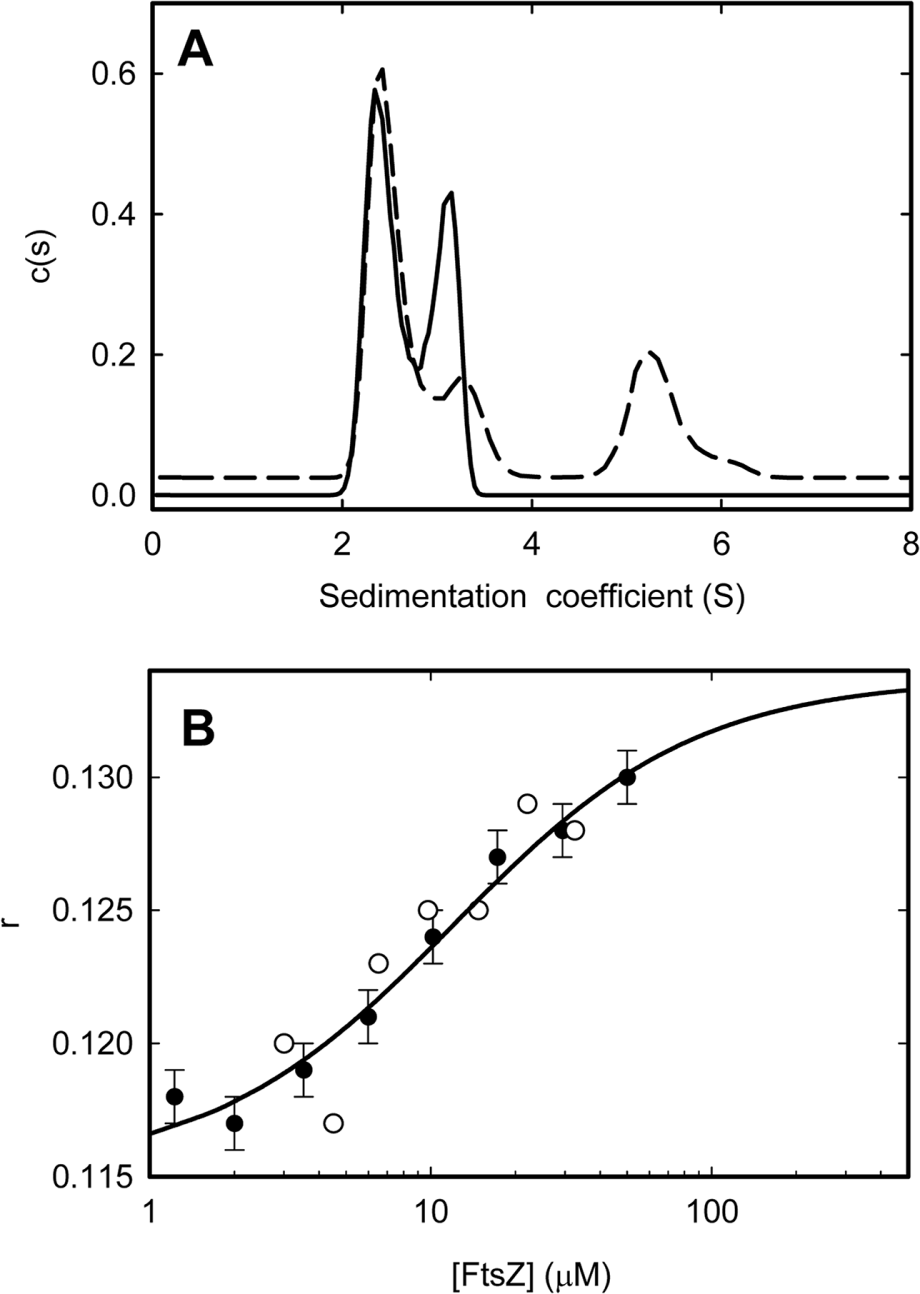
Interaction of SlmA with FtsZ-GDP and FtsZ-GTP: *A*. Sedimentation velocity profiles of FtsZ-GDP (6 μM FtsZ-Alexa 488, 30 μM total FtsZ) in the absence (solid line) or presence of SlmA and SBScons (15 and 4 μM, respectively; dashed line). The profile in the presence of SlmA and SBS has been shifted up 0.025 *c(s)* units for the sake of clarity. *B*. Dependence of the anisotropy (*r*) of fluorescein labeled SBScons (0.2 μM) with SlmA (2 μM) on FtsZ concentration (solid symbols). Results obtained at 10 nM SBScons and 1 μM SlmA are also shown (open symbols). FtsZ polymerization was induced with GTP+RS. Solid line corresponds to the fit of a simple 1:1 model compatible with the data as described in the main text. Isotherm is the average of three independent experiments ± SD. The excitation and emission wavelengths were 495 nm and 520 nm, respectively. Measurements were conducted in working buffer at 20°C.

To get some insight into the mechanism by which SlmA SBS complexes disrupt FtsZ polymers, the ability of the nucleoprotein complexes to bind FtsZ-GDP oligomers and FtsZ-GTP protofilaments was explored. Significant interaction between SlmA SBS and FtsZ-GDP was detected by analytical ultracentrifugation and SEC-MALLS at 300 mM KCl and neutral pH. The sedimentation velocity profiles obtained for FtsZ with FtsZ-Alexa 488 as a tracer displayed two partially overlapping peaks at approximately 2.5 and 3.0 S (**Fig 5A**), consistent with the known tendency of FtsZ-GDP to oligomerize [40]. An additional wide peak around 5.3 S was observed upon addition of SlmA and SBScons (**Fig 5A**), clearly proving the interaction between FtsZ-GDP and SlmA SBS. This interaction was also detected by SEC-MALLS (not shown). The level of interaction found here is compatible with the apparent dissociation constant of approximately 0.2 μM recently reported [35]. Moreover, the concentration of the SlmA SBS complex at which half of the depolymerization effect was achieved (around 0.4–0.5 μM, **Fig 3B**) is close to this affinity value, suggesting that the interactions with FtsZ-GDP oligomers play a key role in SlmA SBS induced disassembly.

SlmA SBS complexes were also found to interact with FtsZ protofilaments elicited by GTP and stabilized by the use of the enzymatic regeneration system. Titration of FtsZ with GTP+RS into solutions containing SlmA and fluorescein-labeled SBScons resulted in an enhancement of the measured fluorescence anisotropy (**Fig 5B**), indicative of interaction of the nucleoprotein complex with FtsZ-GTP protofilaments. Analysis of the isotherm determined at 0.2 μM SlmA SBS to a simple 1:1 model compatible with the data rendered an apparent *K*_*d*_ of 12 μM (*∆G* = 6.6 ± 0.3 kcal/mol). Data obtained at 10 nM SlmA SBS were also compatible with this apparent affinity value (**Fig 5B)**, corresponding to the concentration of FtsZ monomers at which half of the total amount of SlmA SBS complex was bound to FtsZ-GTP. According to data in Fig 3B, at the very low concentration of SlmA SBS used in the titrations (particularly at 10 nM) its effect on the protofilaments appears to be minor. Therefore, it seems reasonable to assume that they remain basically intact under these conditions, and they maintain the average size of around 70 monomers previously determined at 300 mM KCl [11]. The apparent dissociation constant might then be reduced to a value close to that previously obtained for the interaction with FtsZ-GDP (approximately 0.2 μM, [35]). These results point towards a joint contribution of interactions with FtsZ-GTP and FtsZ-GDP species to the overall mechanism through which SlmA SBS antagonize FtsZ assembly.

### SlmA SBS accelerates the disassembly of FtsZ bundles inside permeable GUVs

The influence of SlmA SBS complexes on FtsZ assembly was also analyzed inside GUVs as cell-like compartments in which FtsZ polymerization and functional interactions were previously studied [42,54]. The use of permeable GUVs, containing pores induced in the EPC bilayer by DMPC [43], allows to trigger FtsZ polymerization by the addition of GTP and Mg^2+^ to the medium. Crowding conditions inside GUVs induce the arrangement of protofilaments into observable bundles.

**Fig 6:**
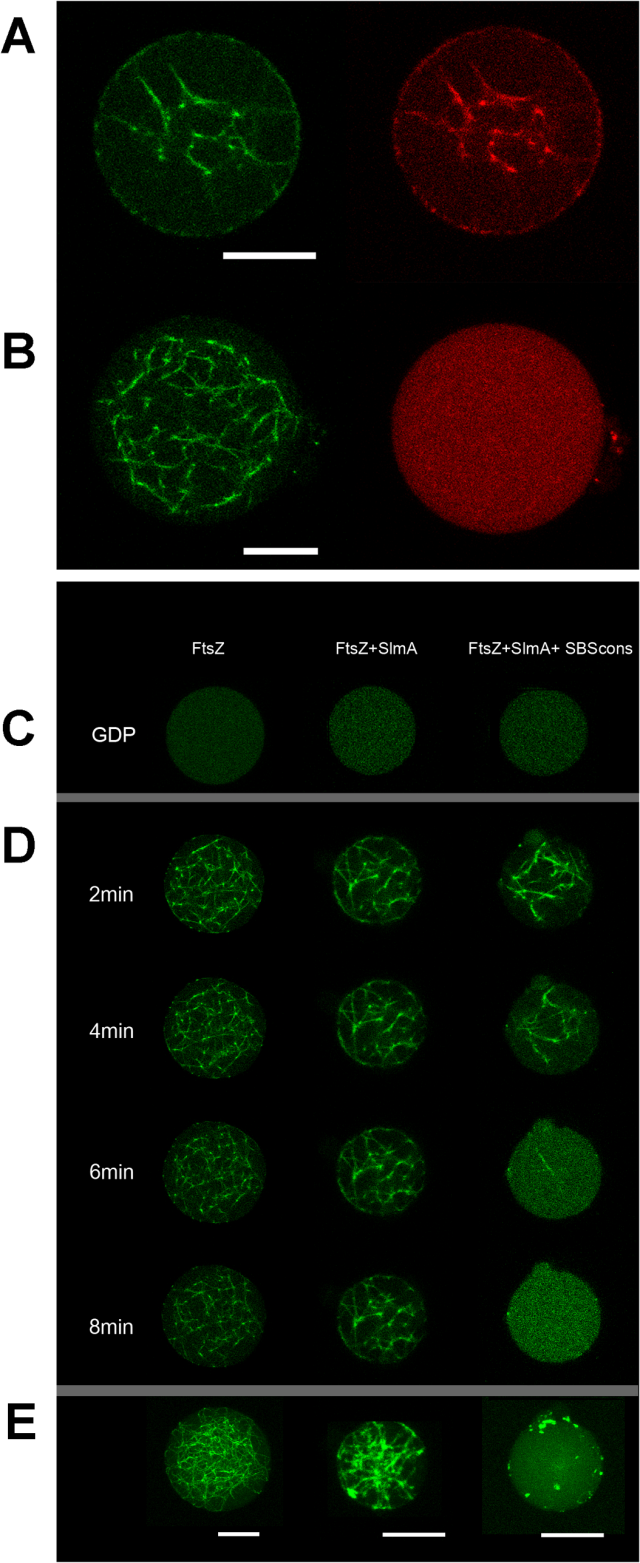
Spatial distribution and lifetime of FtsZ bundles inside GUVs containing SlmA with or without SBS. *A* and *B*. Representative confocal images of the interaction of FtsZ-GTP bundles with SlmA SBS by coencapsulation of FtsZ (1.2 μM FtsZ-Alexa 488, 12 μM total FtsZ) and SlmA (1 μM SlmA-Alexa 647, 10 μM total SlmA) inside GUVs in the absence (*B*) or presence of SBScons (2 μM, *A*). *C*. Representative confocal images of FtsZ, FtsZ/SlmA and FtsZ/SlmA SBScons encapsulated in GUVs before triggering FtsZ polymerization. *D*. Evolution of FtsZ bundles with the specified time in each condition. *E*. Full reconstruction of all confocal sections at timepoint 8 min. Concentrations in *C*-*E* were 10 μM SlmA, 2 μM SBScons, 12 μM FtsZ and 1.2 μM FtsZ-Alexa 488. FtsZ polymerization was induced by adding GTP and magnesium in the solution containing the EPC/DMPC GUVs. Bars correspond to 10 μm.

FtsZ bundles together with functional SlmA SBS complexes were then reconstructed inside permeable GUVs. Coencapsulation of SlmA, SBScons and FtsZ, by addition of the three components to the lipid mixture during vesicle formation, was confirmed by detecting the fluorescence of either protein labeled with Alexa 488 or Alexa 647 (**Fig 6**) or SBS with fluorescein (not shown). Upon addition of GTP, the SlmA SBS complexes seemed to retain the ability to interact with FtsZ bundles in the vesicles, as colocalization of the two proteins labeled with spectrally different dyes was observed (**Fig 6A**). This colocalization was not observed in the absence of SBS and SlmA appeared homogeneously distributed in the vesicle lumen (**Fig 6B**).

As for the protofilaments in solution, SlmA SBS complexes also reduced the lifetime of FtsZ bundles inside GUVs. FtsZ containing tracer amounts of FtsZ-Alexa 488, with or without the nucleoprotein complex, appeared homogeneously distributed in the lumen of the vesicle before triggering polymerization (**Fig 6C**). Diffusion of GTP and Mg^2+^ into the vesicles readily induced the formation of FtsZ filaments inside the GUVs disregarding the presence of SlmA with or without SBS (**Fig 6D**). The variability of the FtsZ polymers inside the GUVs, even in the absence of SlmA, precluded further characterization of the images to ascertain structural changes caused by the nucleoid occlusion factor. However, the presence of the SlmA SBS complex clearly affected the persistence of the FtsZ bundles. After 3–4 minutes, the FtsZ fluorescence signal was dispersed homogeneously throughout the vesicle lumen indicating the release of FtsZ from the bundles (**Fig 6D and 6E**and **S1 Movie**). On the other hand, in the absence of SlmA SBS the GTP-induced FtsZ bundles were stable for over 8–16 min (**Fig 6D and 6E**). GTPase activity measurements in the encapsulation buffer showed a modest enhancement, less than 20%, upon addition of SlmA SBS (from 4.8 to 5.7 moles of hydrolyzed GTP/mole of FtsZ/min). Although this may contribute to the overall effect, it would hardly explain the dramatic reduction of the duration of the polymers by the nucleoprotein complexes. No effect of SlmA in the lifetime of the polymers was observed in the absence of SBS (**Fig 6D and 6E**).

## Discussion

We have investigated the role of the site-selection SlmA protein, which prevents the formation of the division ring in regions close to the nucleoid, on the structural and biochemical properties of FtsZ polymers. Our results show that the functional SlmA unit able to trigger FtsZ disassembly is the 4:1 SlmA SBS complex in which SlmA, as a dimer of dimers, binds DNA. The number of copies of SlmA present in an *E*. *coli* cell (300–400 [36]), relative to the SBS sequences identified (24–52 [31,32]) should be sufficient to allow the binding of at least one SlmA complex to every SBS copy present in the chromosome.

No effect of free SlmA on FtsZ polymers was detected, even at concentrations at which the main oligomeric species of SlmA is a dimer, reinforcing the idea that the interaction of SlmA with SBS is required to assemble the functional dimer of dimers of SlmA able to counteract FtsZ polymerization [34]. Activation of SlmA function upon binding to SBS may result from a conformational change of the protein coupled to DNA binding, as previously suggested [34] or, alternatively, from the joining of two SlmA dimers that, although not interacting with each other, could adopt a different arrangement within the nucleoprotein complex favoring then their inhibitory function.

SlmA, when bound to its specific target DNA sequences (SBSs) accelerates the disassembly of FtsZ protofilaments in solution or FtsZ bundles inside permeable giant vesicles. We find that the interaction of SlmA SBS with FtsZ, in its GTP and GDP bound forms, may break or destabilize the fibers resulting in shorter polymers or oligomers of a variety of sizes able to hydrolyze GTP at the same rate as the intact FtsZ protofilaments. This is in striking contrast to the action of other inhibitors of FtsZ polymerization, including the SOS induced inhibitor SulA [55], and MinC, the inhibitory element of the septum site selection mechanism.

**Fig 7:**
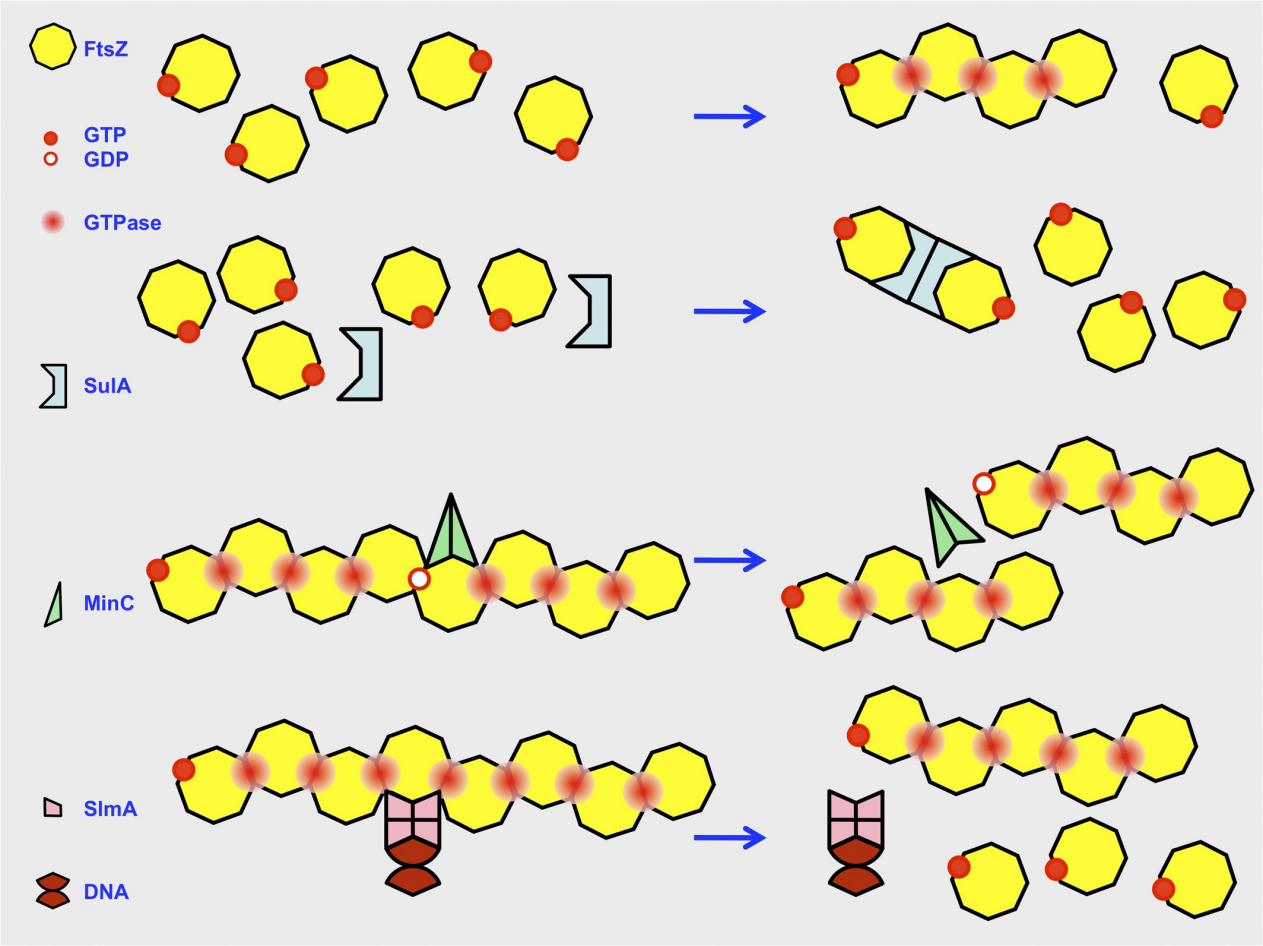
Comparison of the mechanisms used by negative regulators of FtsZ assembly. FtsZ monomers are depicted as having one nucleotide molecule, either GDP or GTP. Polymers form once the concentration of nucleotide-charged monomers reaches a threshold (the critical concentration), arbitrarily fixed here as a minimum of four monomers in a field (top row). FtsZ contained inside polymers displays a GTPase activity. The action of SulA dimers (second row) prevents polymerization by removing two FtsZ monomers from the available pool while locking them in a relative orientation that precludes the assembly of a GTPase active site. As a result the critical concentration is not reached and FtsZ tends to remain as monomers. MinC dimers (third row) interfere with already formed FtsZ filaments dissociating them into shorter polymers of narrowly distributed sizes. SlmA (fourth row) forms dimers of dimers when associated to DNA sequences in the nucleoid. The SlmA SBS complexes dissociate FtsZ polymers yielding shorter polymers of a variety of sizes.

Although both SlmA and MinC disrupt the FtsZ polymers without affecting the GTPase activity of the FtsZ protein ([24,53] and this work), the interaction of MinC with FtsZ in solution does not modify the rate of disassembly. In addition, the action of MinC produces polymers of a narrowly distributed shorter size (**Fig 7**) [53]. Contrary to SlmA SBS, MinC interacts mainly with FtsZ in the GDP bound state [53,56]. This may be a reason why these two proteins act differently on FtsZ filaments. The action of SlmA may be mediated by the formation of stable complexes with the polymers, while MinC more likely disrupts them by interacting with the cycling FtsZ-GDP subunits or by producing transient complexes with the FtsZ-GDP subunits within the polymer [53]. Although MinC and SlmA do not modify the GTPase activity of FtsZ they both require this activity, and the turnover resulting from GTP hydrolysis, for filament disassembly. However, the effect of the two FtsZ inhibitors on FtsZ polymers containing the slowly hydrolysable GMPCPP nucleotide is markedly different. MinC does not modify the GMPCPP-FtsZ polymers [53,57] but intriguingly the presence of this nucleotide increases the bundling promoting activities of SlmA SBS ([35] and this work). MinC and SlmA play correlated roles in selecting the septum placement, it is not so surprising then that even if both act on the polymerization of FtsZ, their action differ in some details to ensure that they are complementary rather than redundant [30].

The concentrations of SlmA SBS required to accelerate FtsZ disassembly are 5 to 10-fold lower than those of FtsZ (1–2 μM vs. 12 μM), while MinC-driven inhibition of FtsZ assembly occurs at equimolar concentrations of MinC and FtsZ [53]. Our findings suggest that the affinity of SlmA SBS for FtsZ is higher than the affinity of the association between MinC and FtsZ, being the estimated apparent dissociation constants 0.2 μM (this work and [35]) and 6–10 μM [53,58], respectively. The binding of MinC to MinD at the membrane results in a 25- to 50-fold enhancement of the inhibitory activity of MinC [25] suggesting that the interaction between MinCD and FtsZ is stronger at the membrane.

The inhibitory action of SlmA SBS on FtsZ polymerization is linked to the binding of SlmA to the FtsZ central hub, a conserved C-terminal tail [35]. This region is involved in a variety of protein-protein interactions, among them with MinC, and the proto-ring elements FtsA and ZipA, besides SlmA. SlmA SBS complexes shorten the lifetime of the FtsZ polymers increasing the number of unassembled FtsZ in the cytoplasm. The unassembled molecules would be available to form polymers in the vicinity of the division sites. Interestingly, FtsA, the proto-ring element responsible for the initial stages of membrane tethering of FtsZ polymers, shares functional similarities to SlmA. At the membrane, FtsA induces the dynamic reorganization of FtsZ polymers that undergo rapid treadmilling [59]. Additionally, FtsA promotes the destabilization of FtsZ polymers, probably by fragmentation, and enhancement of their depolymerization rate. As in SlmA, these FtsA activities do not alter the GTPase activity of FtsZ. These behaviors can be compared to the action of ADF/cofilin, an actin-binding protein that enhances the treadmilling of actin fibers [60] by increasing the rate of polymer disassembly, thus enlarging the size of the monomeric actin reservoir.

The action of SlmA or MinC on FtsZ has a spatial dimension preventing polymerization at undesired places, either the space occupied by the nucleoid or the cell poles respectively. Other FtsZ polymerization inhibitors as SulA may have a temporal inhibitory role to allow sufficient time for DNA repair before division. This may be attained best by a sequestration mechanism preventing altogether FtsZ polymerization until the lesions are mended. Accordingly, SulA blocks FtsZ assembly by sequestration of the FtsZ monomers forming a complex in which a dimer of the inhibitor binds two monomers of FtsZ (**Fig 7**). Besides preventing FtsZ polymerization SulA causes a significant reduction of the FtsZ GTPase activity [55,61,62]. A similar inhibitory mechanism is used by OpgH, a moonlighting glucosyltransferase that coordinates cell size with nutrient availability [63]. When growing in rich medium, the binding of abundant UDP-glucose to OpgH induces a conformational change favoring the sequestration of FtsZ monomers by OpgH and therefore delaying division until a larger size is attained. Decreasing amounts of UDP-glucose revert the binding to OpgH and therefore release FtsZ, allowing division at smaller sizes when growing in minimal media.

As we have described for SlmA, many biochemical details are known on the mechanisms governing the polymerization of FtsZ that seem to act at selecting the proper site for the placement of the FtsZ ring. However, similar details on the properties of the activities of FtsA and ZipA that operate to remodel the FtsZ polymers during constriction, a process that lies at the core of efficient septation in the *E*. *coli* cell, are not so well known and should merit further investigation.

## Supporting Information

S1 Movie Movie reconstructed from the whole collection of images shown in Fig 6, corresponding to the temporal evolution of the FtsZ polymers inside GUVs in the presence of SlmA and SBS. (AVI)
